# Supplementary material for: Ly6Chi Monocytes Are Metabolically Reprogrammed in the Blood during Inflammatory Stimulation and Require Intact OxPhos for Chemotaxis and Monocyte to Macrophage Differentiation
Source: Cells. 2024 May 26;13(11):916. doi: 10.3390/cells13110916 (PMC11171939; doi:10.3390/cells13110916)

Supplementary Figure S1: Full gating strategies for myeloid cells present in the peritoneum.

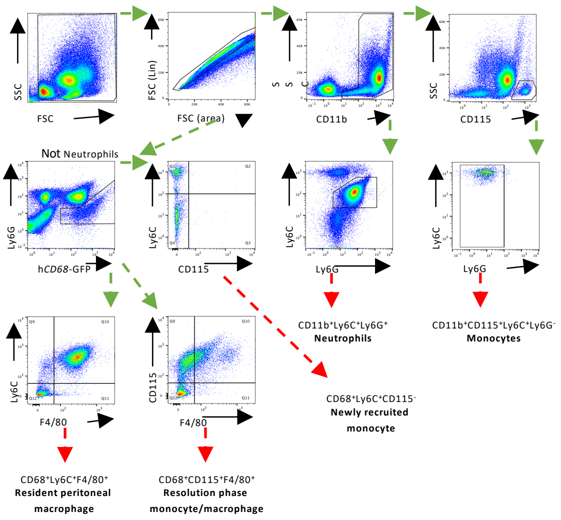

Supplementary Figure S1: Full gating strategies of myeloid cells presents in peritoneum.

Supplementary Figure S2: Adoptive transfer of peritoneal cells following zymosan challenge into ongoing zymosan induced peritonitis.

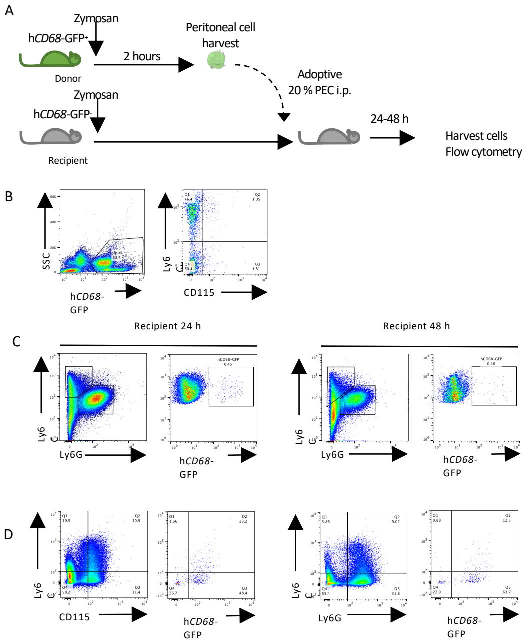

Supplementary Figure S3: Single cell RNA sequencing reveals significant differential gene expression between Ly6C^{hi} monocyte clusters.

A

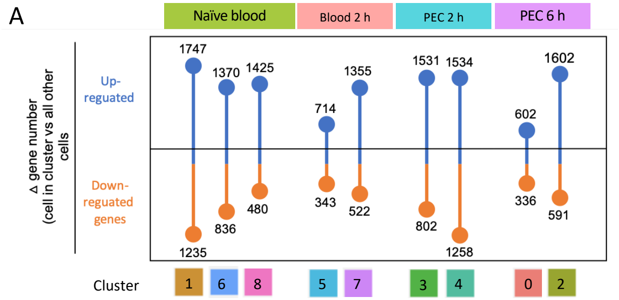

Supplementary Figure S4: Initial quality control of cell hashing and clustering algorithm.

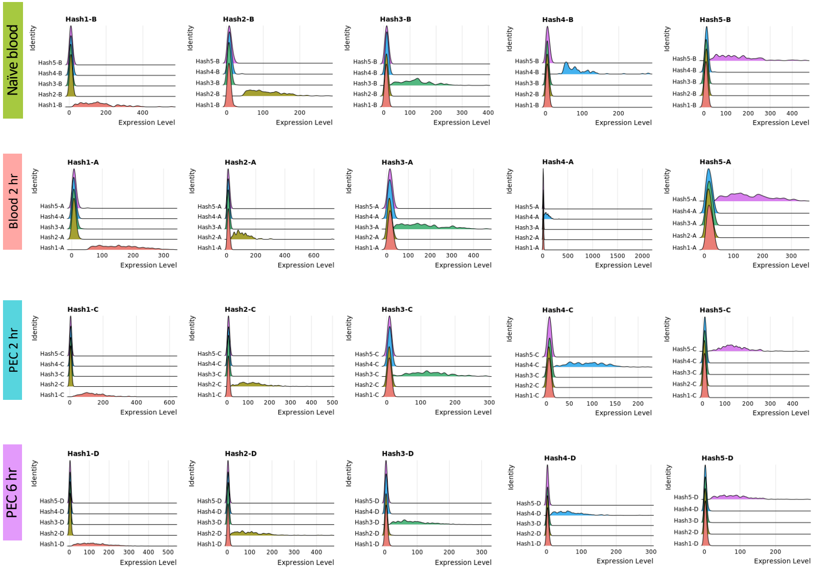

Supplement: Supplementary file 1 [file cells-13-00916-s001.zip › cells-3012229-supplementary.pdf]
